# Supplementary material for: An Experimental Framework for Developing Point-of-Need Biosensors: Connecting Bio-Layer Interferometry and Electrochemical Impedance Spectroscopy
Source: Biosensors (Basel). 2022 Oct 29;12(11):938. doi: 10.3390/bios12110938 (PMC9688365; doi:10.3390/bios12110938)
Supplement: Supplementary file 1 [file biosensors-12-00938-s001.zip › biosensors-1979349-supplementary.pdf]

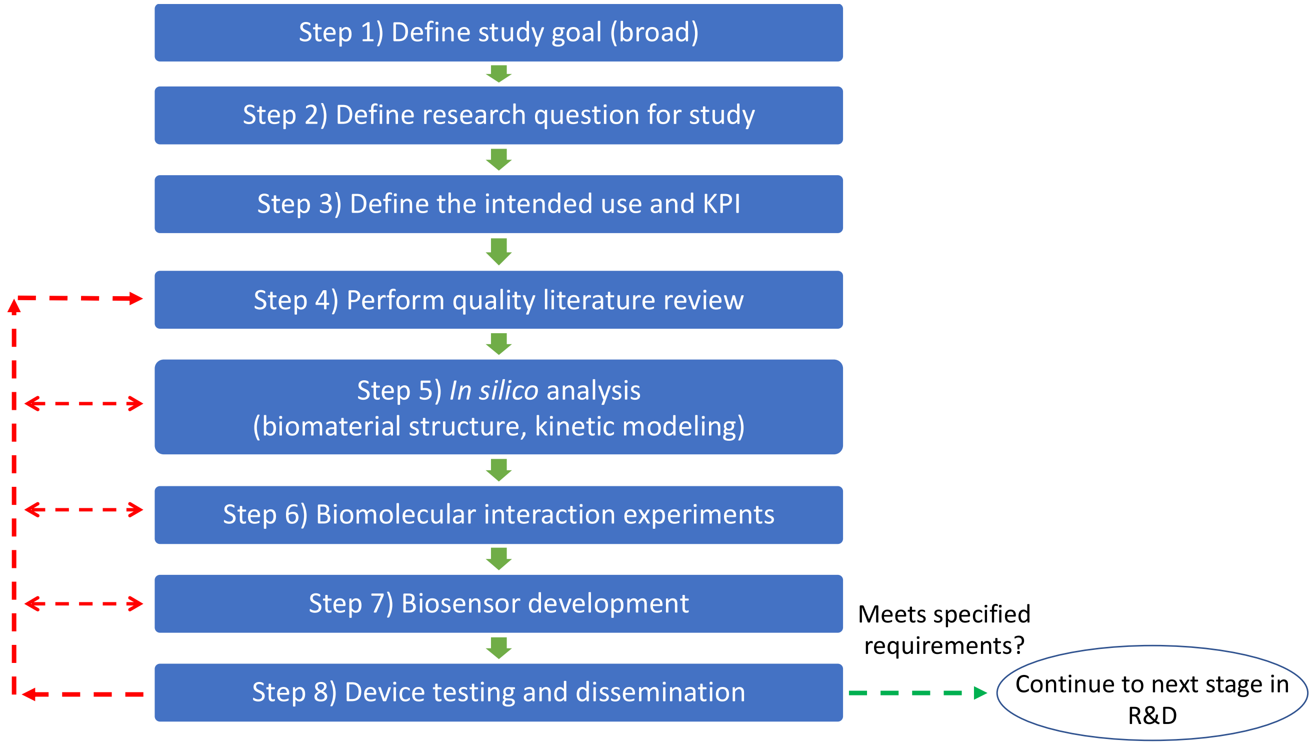

Figure S1. Flowchart for applying framework. Experimental design and iteration in steps 5-7 should always consider the study goal, intended use, and research question of the study as a guide for decision-making. This process also facilitates updating the quality literature review (use of PRISMA framework is highly suggested). Green arrows indicate that the prototype meets the requirements, and we can move to the next process step. Red arrows indicate that the prototype doesn't meet the specified requirements, and an iteration to prior step(s) is required.

1. Goal of Research

Instructions: Describe goal of research (broad in scope).

2. Research question(s)

Instructions: Describe specific research question(s) addressed in this study.

3. Intended use and KPI of proposed device

Instructions: Describe the intended use of the final biosensor (include details of the target audience, if known). Also describe any key performance indicators (KPI), if known. For example, biosensors developed for a particular clinical application are governed by a regulatory agency that sets limits on the percent positive agreement for any proposed diagnostic test.

4. Perform meta-analysis of published literature

Instructions: Use the PRISMA guide to perform a quality literature review on relevant topics. Examples include, but are not limited to, biomaterial structural analysis, interaction kinetics, previous published devices). If applicable, include the flowchart describing the screening process (templates available from PRISMA: https://prisma-statement.org/prismastatement/flowdiagram.aspx). The following steps need to be considered during the literature review process: *a)* Establish screening criteria for literature review; *b)* Create a diagram that shows why articles were screened; *c)* PRISMA as guide and research question as swim lane bumper for screening; *d)* Analyze literature that passes through screening process as defined by PRISMA.

5. Perform in silico analysis

Instructions: Perform analysis (*in silico* or experimental). Examples of analyses may be characterization of biomaterial structure, interaction kinetics or other tasks guided by the goal and research question in steps 1 and 3.

6. Perform biomolecular interaction study

Instructions: Perform analysis (*in silico* or experimental) for characterizing biomaterial(s) used in biosensor development. If experimental work is conducted, an experimental plan and/or experimental design should be included. Ensure that any designed studies satisfy the goal and research question in steps 1 and 3.

7. Perform biosensor development study

Instructions: Develop biosensor and test in controlled conditions. If possible, challenge the biosensor under different anticipated conditions or in field trials. If experimental work is conducted, an experimental plan and/or experimental design should be included. Ensure that any designed studies satisfy the goal and research question in steps 1 and 3.

8. Perform device testing and disseminate results

Instructions: Challenge biosensor in intended application conditions. If possible, communicate results with the target audience directly. At a minimum, disseminate results through appropriate outlets (e.g., peer-reviewed literature, educational programs, patents, etc.). If experimental work is conducted, an experimental plan and/or experimental design should be included. Ensure that any designed studies satisfy the goal and research question in steps 1 and 3.

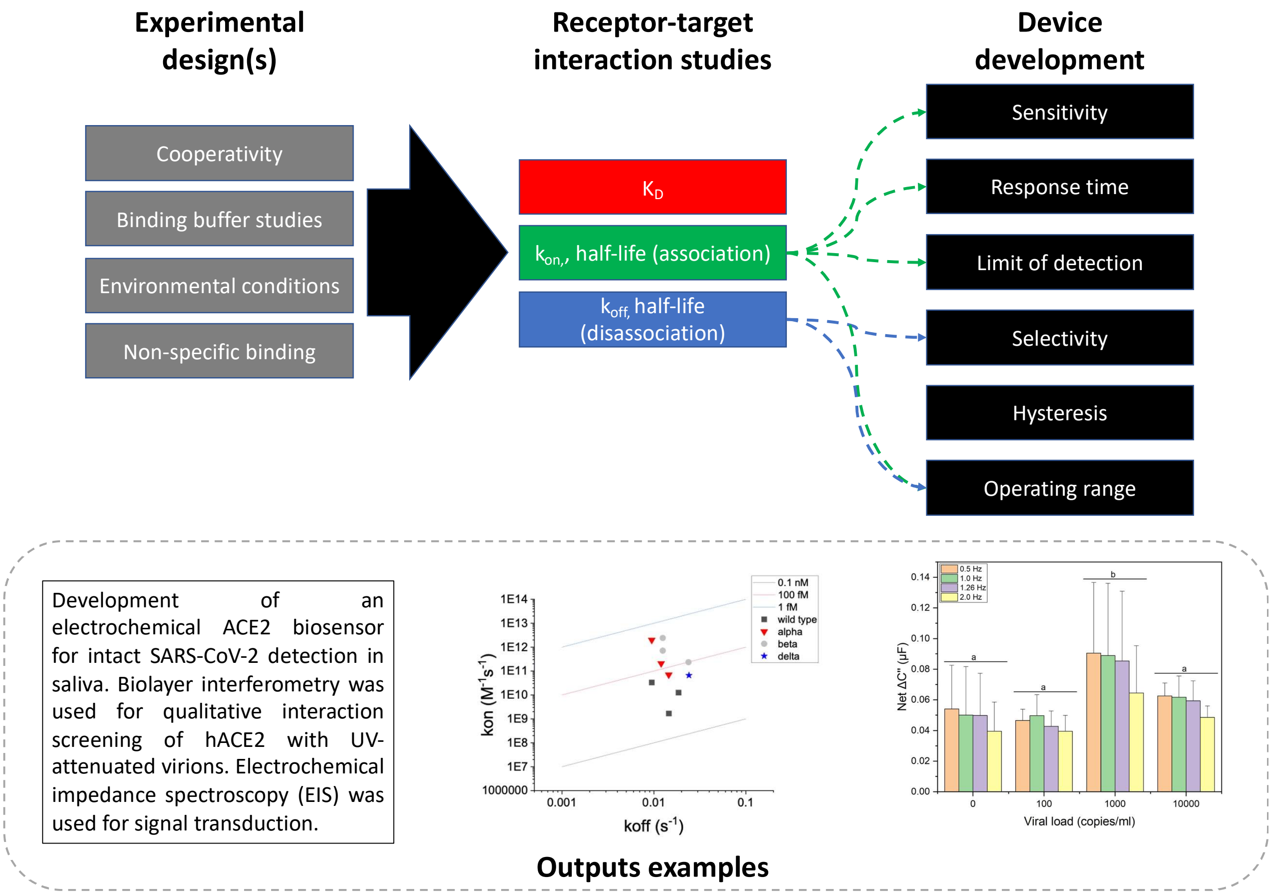

Figure S2. Description of our process and use of the framework in this example.

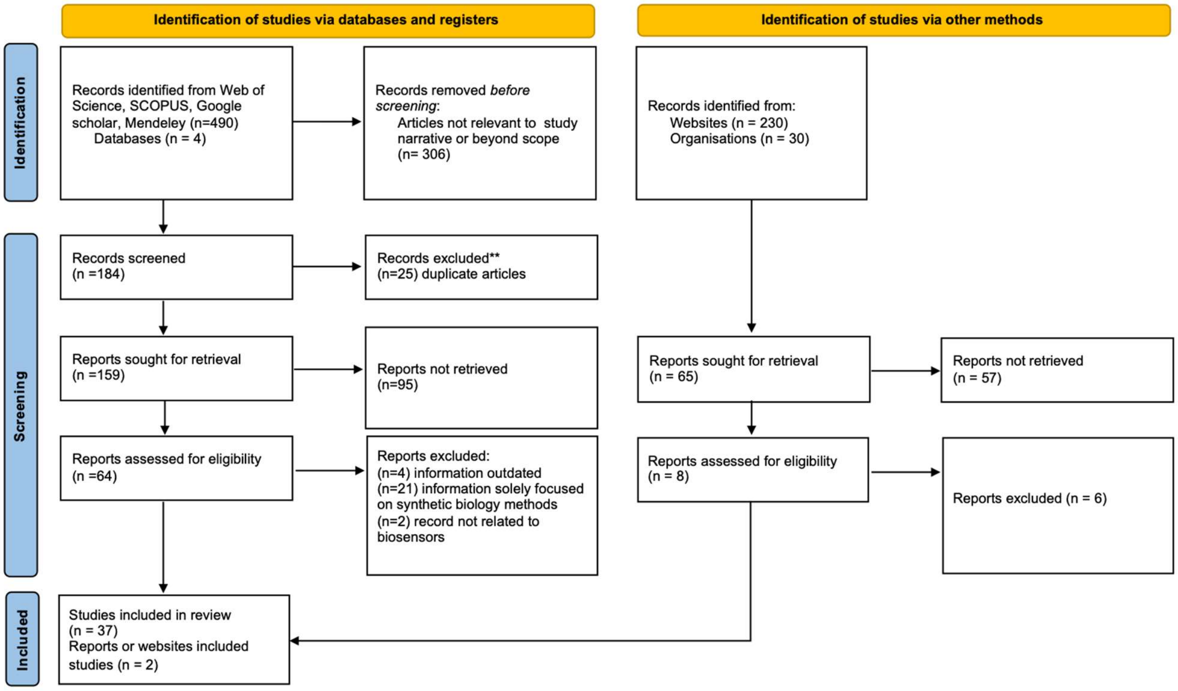

Figure S3. Meta-analysis focused on literature that discusses biomolecular interactions and/or biosensor design.
